# Optimization of culture conditions for differentiation of melon based on artificial neural network and genetic algorithm

**DOI:** 10.1038/s41598-020-60278-x

**Published:** 2020-02-26

**Authors:** Qiang Zhang, Dandan Deng, Wenting Dai, Jixin Li, Xinwen Jin

**Affiliations:** 1 College of Life Science and Technology, Xinjiang University, Ürümqi, China; 2 Institute of Agro-products Processing Science and Technolog, Xinjiang Academy of Agricultural and Reclamation Science, Shihezi, China

**Keywords:** Urogenital models, Tissues

## Abstract

Artificial neural network is an efficient and accurate fitting method. It has the function of self-learning, which is particularly important for prediction, and it could take advantage of the computer’s high-speed computing capabilities and find the optimal solution quickly. In this paper, four culture conditions: agar concentration, light time, culture temperature, and humidity were selected. And a three-layer neural network was used to predict the differentiation rate of melon under these four conditions. Ten-fold cross validation revealed that the optimal back propagation neural network was established with traingdx as the training function and the final architecture of 4-3-1 (four neurons in the input layer, three neurons in the hidden layer and one neuron in the output layer), which yielded a high coefficient of correlation (*R*^2^, 0.9637) between the actual and predicted outputs, and a root-mean-square error (RMSE) of 0.0108, suggesting that the artificial neural network worked well. According to the optimal culture conditions generated by genetic algorithm, tissue culture experiments had been carried out. The results showed that the actual differentiation rate of melon reached 90.53%, and only 1.59% lower than the predicted value of genetic algorithm. It was better than the optimization by response surface methodology, which the predicted induced differentiation rate is 86.04%, the actual value is 83.62%, and was 2.89% lower than the predicted value. It can be inferred that the combination of artificial neural network and genetic algorithm can optimize the plant tissue culture conditions well and with high prediction accuracy, and this method will have a good application prospect in other biological experiments.

## Introduction

Plant tissue culture is a collection of techniques used for studying plant growth, differentiation, gene function and genetic recombination, also an important method for breeding and rapid propagation of crops^1,2^, and thus has been widely used in plant research and agricultural production. As a common technique for plant production, tissue culture is easy to operate, without the need for high-precision instruments. However, there are still some shortcomings in plant tissue culture, such as cumbersome experimental steps and long cycle. During the whole tissue culture experiment, callus induction and organ differentiation are the critical steps where the explants dedifferentiate and form calluses, and then differentiate into plantlets. The calluses undergo a complicated process to differentiate into a variety of plant tissues and organs, and many problems may occur during this process^3^. So, suitable tissue culture conditions are crucial for callus induction and differentiation process. It will be a tedious and time-consuming process to explore specific tissue culture conditions for each plant species due to the large number of plant species. Therefore, the parameters for the model plants such as *Arabidopsis thaliana* and tobacco are adopted in most tissue culture experiments. However, the optimal hormone concentration^4^, culture temperature, humidity, light intensity and duration for *in vitro* culture differ among plant species^5,6^, and vitrification and browning often happen under these unsuitable conditions, and subsequently the entire experiment may fail^7,8^. Therefore, it is of great significance to develop a simple method to rapidly optimize the tissue culture conditions for different plants and to improve the overall efficiency of tissue culture experiments.

Artificial neural networks (ANNs) are developed to deal with noisy, incomplete data and nonlinear problems^9^. ANNs have the ability to identify and approximate any complex nonlinear systems, by which mathematical models can be established rapidly with limited experimental data^10,11^. Moreover, ANNs are more accurate than other common fitting methods (such as response surface methodology)^12^. Training function and the number of neurons in the hidden layer are two factors that directly affect the performance of neural networks^13^. Studies have shown that 10-fold cross validation is a good choice to provide error estimates^14^, and thus can be used to determine the optimal training function and the optimal number of neurons in the hidden layer of neural networks.

Genetic algorithm is a search algorithm used to solve optimization in computational mathematics, it was mostly wide used evolutionary algorithms (EA) algorithms. Genetic algorithms (GAs) are adaptive heuristic search algorithms premised on the evolutionary ideas of natural selection and genetic, and have been extensively used in combination with ANNs for solving optimization problems^15^. Genetic algorithm belongs to the family of meta heuristic algorithms. Metaheurisics can be further divided into two groups: those that are evolutionary algorithms (EA) and swarm intelligence^16^. The most prominent representatives of nature-inspired metaheuristics are evolutionary algorithms (EA) and swarm intelligence^17,18^.

GA is the most well-known representatives of EA, and swarm intelligence are inspired by the social and cooperative behavior of ants, bees, birds, fish, etc^19^.

One of the most relevant characteristics of group system is that individual agents show intelligent behavior together, without the central component to coordinate and guide their activities^20^.

GA can be used in combination with other algorithms to produce better results. GI-ABC, ABC were modified based on genetic algorithm (GA) operators and were applied to the creation of new candidate solutions, which improves the performance of the ABC algorithm by applying uniform crossover and mutation operators from genetic algorithms^21^.

GA proved to be capable of solving large number of NP hard problems also, including problems from the domain of WSNs^22^. Sharma, G etc proposed a distributed range-free node localization algorithm for three dimensional WSNs based on the GA^23^. Similarly, by applying the localization algorithm that employs GA, the localization accuracy of unknown nodes in WSNs was improved^24^, and a novel range free localization algorithm based on GA and connectivity was proposed recently also^25^.

Naturally inspired algorithms have been successfully used in combination with other ANNs, especially with Convolutional Neural Networks (CNN). Convolutional neural network (CNN) is a kind of special deep neural network. CNN have proved to be a robust method for tackling various image classification tasks^16^, and has been widely used in the field of computer vision in recent years^26^. GA have many successful applications in the domains of deep learning and CNNs^17,18^. Better results can be obtained by applying meta-heuristic methods such as genetic algorithm (GA)^17^ and swarm intelligence^27^ to the process of CNN hyperparameter optimization.

In this study, the differentiation of tissue-cultured melon was induced under different conditions (agar concentration, relative humidity, culture temperature and light duration were set at three levels each), and a three-layer neural network was used to predict the non-linear relationship between the culture conditions and differentiation rate, based on which a GA was used for global optimization to determine the optimal combination of culture conditions.

## Results

### CCD result

The rate of differentiation measured by CCD experiment and that predicted using ANN were represented in Table 1.

**Table 1:** Show the rate of differentiation measured by CCD experiment and that predicted using ANN.

No.	Y_1_	Y_2_	Y_3_	Y_4_	The rate of differentiation (*P*, %)	
					Actual values	Predicted values
1	−1	−1	−1	−1	83.21	82.89
2	−1	−1	−1	1	80.37	80.26
3	−1	−1	1	−1	76.53	77.35
4	−1	−1	1	1	74.82	76.15
5	−1	1	−1	−1	80.15	81.83
6	−1	1	−1	1	76.38	76.28
7	−1	1	1	−1	73.75	73.66
8	−1	1	1	1	69.85	69.88
9	1	−1	−1	−1	87.82	87.35
10	1	−1	−1	1	85.38	82.54
11	1	−1	1	−1	83.34	83.23
12	1	−1	1	1	80.52	79.97
13	1	1	−1	−1	85.5	85.62
14	1	1	−1	1	82.99	81.63
15	1	1	1	−1	80.33	80.85
16	1	1	1	1	76.51	76.74
17	−2	0	0	0	76.29	75.87
18	2	0	0	0	78.52	80.11
19	0	−2	0	0	82.59	84.84
20	0	2	0	0	78.38	79.83
21	0	0	−2	0	78.43	81.47
22	0	0	2	0	71.87	71.38
23	0	0	0	−2	80.26	79.69
24	0	0	0	2	76.37	76.19
25	0	0	0	0	82.98	83.16
26	0	0	0	0	83.25	83.16
27	0	0	0	0	83.06	83.16
28	0	0	0	0	83.32	83.16
29	0	0	0	0	82.65	83.16
30	0	0	0	0	83.29	83.16
31	0	0	0	0	83.37	83.16
RMSE						0.0108
*R*^2^						0.9637

### Neural network

**Figure 1:**
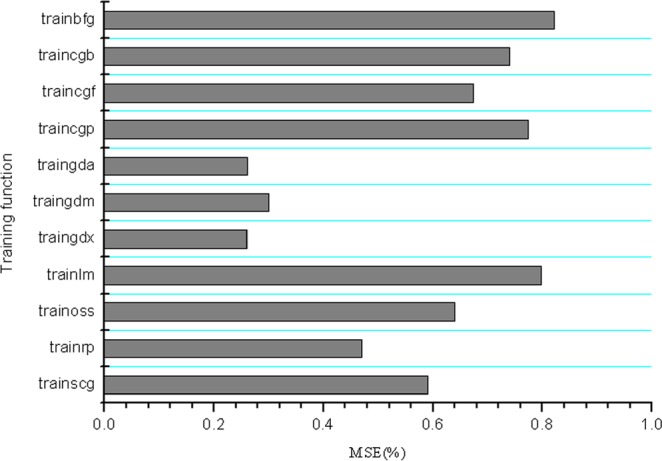
Comparison of the 11 BP algorithms with 6 neurons in the hidden layer.

Artificial neural network is able to inversely regulate the weights and thresholds of the neurons of the input layer by comparing the difference between the actual output and the predicted output, to minimize the overall error^28^. As shown in (Fig. 1), five-fold cross validation revealed that the minimum MSE value was achieved with traigdx (a network training function that updates weight and bias values according to gradient descent momentum and an adaptive learning rate) among the 11 training functions, when there were six neurons in the hidden layer. Therefore, traingdx was considered as the optimal training function of the neural network we established.

About training function “traingdx”: when BP neural network is trained, learning speed too fast may cause instability, too slow it will take too much time, and different training algorithms also have a great impact on the performance of the network. Some studies believe that “trainlm” is more suitable for fitting functions. Indeed, using “trainlm” can obtain higher model accuracy than “traingdx”. However, once the test data is used for simulation, the error between simulation output value and real value is higher than “traingdx” method. As shown in Fig. 1, the error between simulation output value and real value of test data cannot be reduced by “trainlm”. In this paper, the “traingdx” with a slow learning rate is used as the training method. And the average error between the simulation output value and the real value is the smallest. Therefore, “traingdx” is used as the training function. Although “traingdx” has the problems of slow learning speed and long training time, the training time of the model is not considered because of the small sample size of the data in this paper.

**Figure 2:**
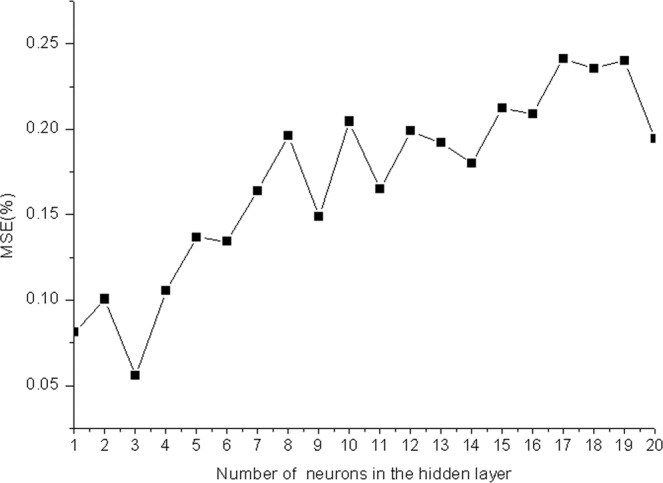
Relationship between number of neurons in the hidden layer and MSE.

Then, the function was used to perform ten-fold cross validation to determine the optimal number of neurons in the hidden layer. As shown in (Fig. 2), the MSE value between the actual and predicted outputs decreased dramatically at first with the number of hidden layer neurons increasing from 1 to 20, but then decreased slightly with the number of hidden layer neurons increasing from eight to thirteen. Because the non-linearity between the factors selected in the experiment and the response value is weak, if the number the hidden layer and neurons were too much, the structure of the BP neural network will be complicated, and the training time will be prolonged. What’s more important is that the model will be over fitting, reducing the generalization ability of the model, and reducing the prediction accuracy. As shown in the Fig. 2, when the number of hidden layer neurons is 3, the average error between the simulated output value of the test data and the true value is the smallest.

So, 3 was considered as the optimal number neurons in the hidden layer. In summary, the optimal backpropagation neural network was established with traingdx as the training function and the final architecture of 4-3-1 (four neurons in the input layer, 3 neurons in the hidden layer and one neuron in the output layer), which yielded a high coefficient of correlation (*R*^2^, 0.9637) between the actual and predicted outputs, and a RMSE value of 0.0108, as shown in Table 1, indicating that the neural network worked well and achieved a high accuracy of prediction.

### Optimal culture conditions for differentiation of melon produced by the genetic algorithm

**Figure 3:**
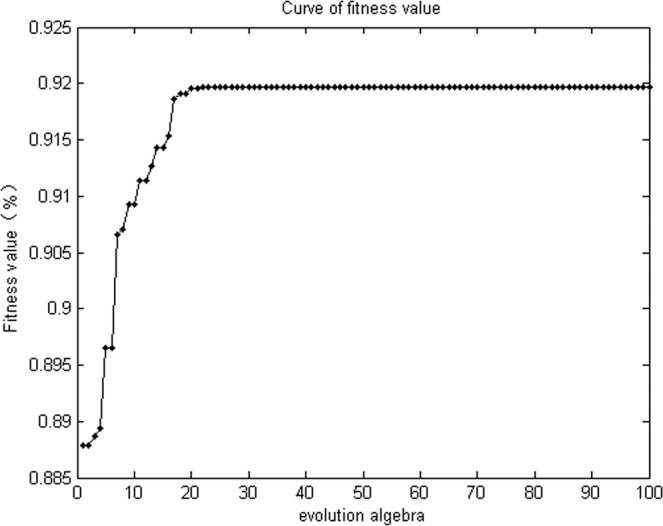
Curve of fitness value per generation of the genetic algorithm.

As shown in (Fig. 3), over 15 generations, the fitness value of the genetic algorithm approached towards the maximum 91.97%, which was the maximum predicted differentiation rate, and could be achieved under the culture conditions as follows: agar concentration of 0.8%, light duration of 8 h/d, culture temperature of 20°C and humidity of 58.85%.

### Validation of optimal culture conditions

The optimal culture conditions produced by the genetic algorithm were then verified by tissue culture experiment. As shown in Table 2, the actual differentiation rate of the three replicates in the experiment was 90.53% on average, which was only 1.44% lower than the predicted value, suggesting that the optimal culture conditions produced by the genetic algorithm are reliable and feasible.

**Table 2:** Show the comparison between GA predicted and actual differentiation.

Replicates	GA predicted value (%)	Actual value (%)
1	91.97	90.35
2		89.97
3		91.26
Mean		90.53
Error (%)		1.59

To predict the differentiation rate of melon under different culture conditions, a BP neural network was established with traingdx as the training function and the final architecture of 4-3-1 in the present study, which yielded a high coefficient of correlation (*R*^2^, 0.9637) between the actual and predicted outputs, and a RMSE value of 0.4971, indicating that the artificial neural network meets statistical requirements. The optimal culture conditions for differentiation induction of melon produced by the genetic algorithm were agar concentration of 0.8%, light duration of 8 h/d, culture temperature of 20 °C and humidity of 58.85%. Under these conditions, the differentiation rate of melon was improved to 90.53%, which was 1.59% lower than GA-predicted value (91.97%), but about 22.66% higher than the differentiation rate (74.98%) achieved under previous culture conditions. The results proved that artificial neural networks combined with genetic algorithms are able to optimize the tissue culture conditions of plants. Similarly, this method can also be used to optimize other stages of tissue culture and rapid propagation conditions of plants *in vitro*.

### Comparison with the optimization by response surface methodology

The optimal culture conditions for differentiation induction of melon produced by response surface methodology of the CCD design method of the Design-Expert software were the agar concentration of 0.68%, light duration of 10 h/d, culture temperature of 25.5 °C and humidity of 64.22%, with the correlation (*R*^2^, 0.8751), and the predicted differentiation rate was 86.04%. Under this predicted conditions, as shown in Table 3, the actual differentiation rate of melon was to 83.62%, which was 2.89% lower than predicted value. These results indicating that the neural network was better than the response surface methodology in this experiment.

**Table 3:** Show the comparison between response surface methodology predicted and actual differentiation.

Replicates	GA predicted value (%)	Actual value (%)
1	86.04	83.85
2		82.65
3		84.37
Mean		83.62
Error (%)		2.89

## Discussion

Many genes expressions in plants are affected by environmental factors stress. This method can also optimize the selected stress factors, regulate the expression of target genes more effectively, which would be helpful to the study of genes function. In addition, a lot of the functional components of medicinal plants are plant secondary metabolites, which have been widely used in medicine. Some productions and accumulation of plant secondary metabolites are mostly affected by environmental factors^29^. For example, many medicinal plants have great differences in their medicinal properties when the plants grown under different environments^30^. Using this method, we can quickly and accurately obtain some medicinal plants growth conditions which conducive to the productions and accumulation of functional components.

We have developed a fast and accurate method for optimizing plant tissue culture conditions. This method is flexible to use, the experimenter can set the condition factors and levels according to the actual situation, and use the optimization algorithm model to optimize the condition parameters quickly, thereby achieving the purpose of improving the experimental success and saving the experiment time.

## Materials and Methods

### Callus induction

The seeds of a melon cultivar Jiashi were shelled manually, sterilized and inoculated onto MS medium. The cotyledons that began to turn green were collected, cut into small pieces of 5 mm long and 5 mm wide, plated onto preculture medium (MS + 1.0 mg/L 6-BA + 0.1 mg/L NAA), cultured first in the dark for 48 h, and then under a 16-h light/ 8-h dark photoperiod until callus formation was observed.

### Differentiation induction

The calluses were transferred onto differentiation medium (MS + 1.0 mg/L 6-BA + 0.1 mg/L IAA), and cultured under different conditions, designed with a five-level four-factor central composite design (CCD)^31^. The independent variables were agar concentration, light duration, culture temperature and relative humidity. The variables and their levels for the CCD were represented in Table 4.

**Table 4:** Show the light duration, culture temperature and relative humidity and their levels for the CCD.

Independent variables	Levels				
	−2	−1	0	1	2
Agar concentration (Y_1_, %)	0.4	0.5	0.6	0.7	0.8
Light duration (Y_2_, h/d)	8	10	12	14	16
Culture temperature (Y_3_, °C)	20	24	28	32	36
Relative humidity (Y_4_, %)	50	60	70	80	90

### Determination of differentiation rate

Differentiation rate was calculated using this formula:

$$P=\frac{{\omega }_{0}\,-\,{\omega }_{1}}{{\omega }_{0}}\times 100 \% $$

where, *P* is the differentiation rate, *ω*_0_ is the number of inoculated calluses; *ω*_1_ is the number of calluses that differentiated into plantlets.

### Design of neural network architecture

A three-layer backpropagation (BP) neural network was developed with Matlab 7.0 (The MathWorks, Inc., USA), with the factors that affect differentiation induction of melon as the inputs, and the differentiation rate as the output. The tan-sigmoid transfer function tansig was used in the hidden layer, and the linear transfer function purelin in the output layer. The architecture of the BP neural network was designed as follows:

### Ten-fold cross validation

The BP neural network was trained based on the experimental data obtained from CCD, using 11 different training functions, and the number of neurons in the hidden layer was set between 1 and 15. A ten-fold cross validation approach was used to determine the optimal training function of the neural network and the optimal number of neurons in the hidden layer, and the mean square error (MSE)-the average squared error between the network outputs and the target data was used to evaluate the prediction accuracy of the network. In order to improve the stability and accuracy of prediction, the cross-validation was repeated ten times and results were averaged. Mean square error (MSE) was defined as follows:

$$MSE=\frac{1}{n}\mathop{\sum }\limits_{i}^{n}{({P}_{i}-{\hat{P}}_{i})}^{2}$$

where, *P*_*i*_ is target data (actual differentiation rates), ${\hat{P}}_{i}$ is network outputs (predicted differentiation rates), and *n* is the number of target data.

### Evaluation of prediction accuracy of the neural network

The prediction accuracy of the neural network was evaluated by the correlation coefficient (*R*^2^) and root-mean-square error (RMSE) between the network outputs and target data. The correlation coefficient (*R*^2^) was calculated using the formula as follows:

$${R}^{2}={(\frac{n\mathop{\sum }\limits_{i=1}^{n}{P}_{i}{\hat{P}}_{i}-\mathop{\sum }\limits_{i=1}^{n}{P}_{i}\cdot \mathop{\sum }\limits_{i=1}^{n}{\hat{P}}_{i}}{\sqrt{n\mathop{\sum }\limits_{i=1}^{n}{{P}_{i}}^{2}-{(\mathop{\sum }\limits_{i=1}^{n}{P}_{i})}^{2}}\cdot \sqrt{n\mathop{\sum }\limits_{i=1}^{n}{{\hat{P}}_{i}}^{2}-{(\mathop{\sum }\limits_{i=1}^{n}{\hat{P}}_{i})}^{2}}})}^{2}$$

where, *P*_*i*_ is target data (actual differentiation rates), ${\hat{P}}_{i}$ is network outputs (predicted differentiation rates), and *n* is the number of experimental data.

Root-mean-square error (RMSE) was calculated using the formula as follows:

$$RMSE=\sqrt{\frac{1}{n}\mathop{\sum }\limits_{i}^{n}{({P}_{i}-{\hat{P}}_{i})}^{2}}$$

where, *P*_*i*_ is target data (actual differentiation rates), ${\hat{P}}_{i}$ is network outputs (predicted differentiation rates), and *n* is the number of target data.

### Optimization using genetic algorithm

According to the relationship of differentiation rate with agar concentration, light duration, culture temperature and humidity was established according to the BP neural network. The trained neural network was used as a fitness function of the genetic algorithm. The optimization variables were represented as floating-point numbers. The genetic algorithm was run by setting the initial population size at 20, crossover probability at 0.8, and the maximum number of iterations at 100. The prediction accuracy of the genetic algorithm was evaluated by the relative error between the GA predicted data and the actual experimental data, which was calculated using the formula as follows:

$$E( \% )=\frac{|P{\prime} -P|}{P}\times 100$$

where, *P’* is the differentiation rate predicted using GA, and *P* is the actual differentiation rate measured in tissue culture experiment.

## References

[1] Guangchu Z, Yuxia W, Yuanjie T, Xingwei L (2004). *In-vitro* rapid propagation of clumping bamboos. Journal of Bamboo Research..

[2] Hong C, Guoen H, Jiangping F (2013). *In vitro* rapid propagation of wild cherry in Guizhou Province. Jiangsu Agricultural Sciences..

[3] Hongbing G, Xin Q, Huan W, Xiaojie T (2007). Factors affecting vitrification of *Prunus cerasus* cultured *in vitro*. Journal of Anhui Agricultural Sciences.

[4] Yuying Z, Yonghui L, Congyu L, Hongwei G, Shubing C (2007). Influences of 6-BA, NAA and IAA on cotyledons differentiation of cucumber *in vitro*. Journal of Yangtze University..

[5] Guiping R, Xiaojing W, Genfa Z (2016). Effect of LED in different light qualities on growth of *Phalaenopsis* plantlets. Chinese Bulletin of Botany..

[6] Jian C, Zhiwei H (2016). Effects of different media formulations on the rooting of tissue cultured seedlings of *Bletilla striata*. Journal of Sichuan Forestry Science and Technology..

[7] Manzhi S, Chengtao Y (2001). Occurrence and prevention of vitrification during plant tissue culture. Journal of Shandong Forestry Science and Technology..

[8] Juan P, Xianyuan L, Mingyang L (2009). Frequent problems in plant tissue culture and countermeasures. Journal of Anhui Agricultural Sciences..

[9] Morris AJ, Montague GA, Willis MJ (1994). Artificial neural networks: studies in process modeling and control. Trans I Chem Eng..

[10] Pareek VK, Brungs AA, Sharma R (2002). Artificial neural network modeling of a multiphase photodegradation system. Journal of Photochemistry and Photobiology A: Chemistry..

[11] AleDabbous AN, Kumar P, Khan AR (2017). Prediction of airborne nanoparticles at roadside location using a feedforward artificial neural network. Atmospheric Pollution Research..

[12] He L, Xu YQ, Zhang XH (2008). Medium factor optimization and fermentation kinetics for phenazine-1-carboxylic acid production by Pseudomonas sp. M18G. Biotechnology and Bioengineering..

[13] Elmolla ES, Chaudhuri M, Eltoukhy MM (2010). The use of artificial neural network (ANN) for modeling of COD removal from antibiotic aqueous solution by the Fenton process. Journal of hazardous materials..

[14] Fudi C, Hao L, Zhihan X, Shixia H, Dazuo Y (2015). User-friendly optimization approach of fed-batch fermentation conditions for the production of iturin A using artificial neural networks and support vector machine. Electronic Journal of Biotechnology..

[15] Witten I.H. & Frank E. Data Mining: Practical machine learning tools and techniques, Beijing: China Machine Press. 286 (2006).

[16] Strumberger E, Tuba N, Bacanin M, Beko M (2019). Tuba. Convolutional Neural Network Architecture Design by the Tree Growth Algorithm Framework. International Joint Conference on Neural Networks (IJCNN).

[17] Young SR, Rose DC, Karnowski TP, Lim S-H, Patton RM (2015). Optimizing deep learning hyper-parameters through an evolutionary algorithm. Proceedings of the Workshop on Machine Learning in High-Performance Computing Environments, MLHPC..

[18] Ijjina EP, Chalavadi KM (2016). Human action recognition using genetic algorithms and convolutional neural networks. Pattern Recognition.

[19] Yang X-S (2014). Swarm intelligence based algorithms: a critical analysis. Evolutionary Intelligence.

[20] Del Ser, J. Geem, Z. W. & Yang, X.-S. Foreword: New theoretical insights and practical applications of bio-inspired computation approaches. Swarm and Evolutionary Computation, **45**, 10.1016/j.swevo.2018.12.008 (2019).

[21] Bacanin N, Tuba M (2012). Artificial Bee Colony (ABC) Algorithm for Constrained Optimization Improved with Genetic Operators. Studies in Informatics and Control.

[22] Strumberger I, Minovic M, Tuba M, Bacanin N (2019). Performance of Elephant Herding Optimization and Tree Growth Algorithm Adapted for Node Localization in Wireless Sensor Networks. Sensors.

[23] Sharma G, Kumar A (2018). Improved range-free localization for three-dimensional wireless sensor networks using genetic algorithm. Comput. Electr. Eng..

[24] Peng B, Li L (2015). An improved localization algorithm based on genetic algorithm in wireless sensor networks. Cognit. Neurodyn..

[25] Najeh T, Sassi H, Liouane N (2018). A Novel Range Free Localization Algorithm in Wireless Sensor Networks Based on Connectivity and Genetic Algorithms. Int. J. Wirel. Inf. Netw..

[26] Jmour N, Zayen S, Abdelkrim A (2018). Convolutional neural networks for image classification. International Conference on Advanced Systems and Electric Technologies.

[27] Qolomany B, Maabreh M, Al-Fuqaha A, Gupta A, Benhaddou D (2017). Parameters optimization of deep learning models using particle swarm optimization. International Wireless Communications and Mobile Computing Conference (IWCMC).

[28] Hagan, M. T., Demuth, H. B. & Beale, M. H. Neural network design, Beijing: China Machine Press. 127–128 (2002).

[29] Verma N, Shukla S (2015). Impact of various factors responsible for fluctuation in plant secondary metabolites. J Appl Res Med Aromat Plants..

[30] Li Y, Wu. H (2018). The Research Progress of the Correlation Between Growth Development and Dynamic Accumulation of the Effective Components in Medicinal Plants. Chinese Bulletin of Botany..

[31] Nagata Y, Chu KH (2003). Optimization of a fermentation medium using neural networks and genetic algorithms. Biotechnology Letters.

